# Starvation Induces Proteasome Autophagy with Different Pathways for Core and Regulatory Particles*

**DOI:** 10.1074/jbc.M115.699124

**Published:** 2015-12-15

**Authors:** Kenrick A. Waite, Alina De-La Mota-Peynado, Gabrielle Vontz, Jeroen Roelofs

**Affiliations:** From the Division of Biology, Kansas State University, Manhattan, Kansas 66506

**Keywords:** autophagy, deubiquitylation (deubiquitination), proteasome, protein translocation, vacuole, yeast, nitrogen starvation, proteaphagy, ribophagy

## Abstract

The proteasome is responsible for the degradation of many cellular proteins. If and how this abundant and normally stable complex is degraded by cells is largely unknown. Here we show that in yeast, upon nitrogen starvation, proteasomes are targeted for vacuolar degradation through autophagy. Using GFP-tagged proteasome subunits, we observed that autophagy of a core particle (CP) subunit depends on the deubiquitinating enzyme Ubp3, although a regulatory particle (RP) subunit does not. Furthermore, upon blocking of autophagy, RP remained largely nuclear, although CP largely localized to the cytosol as well as granular structures within the cytosol. In all, our data reveal a regulated process for the removal of proteasomes upon nitrogen starvation. This process involves CP and RP dissociation, nuclear export, and independent vacuolar targeting of CP and RP. Thus, in addition to the well characterized transcriptional up-regulation of genes encoding proteasome subunits, cells are also capable of down-regulating cellular levels of proteasomes through proteaphagy.

## Introduction

The degradation of proteins in eukaryotic cells is largely mediated by two machineries, the lysosome (or vacuole in yeast and plants) and the proteasome. The lysosome is a membrane-surrounded organelle that houses numerous acidic hydrolases. These hydrolases can degrade a large variety of substrates, including soluble proteins, aggregates, structured complexes, and even whole bacteria. Entry into the lysosome or vacuole normally requires substrates to be packaged as vesicular material. This includes, among others, endosomes, multivesicular body-derived material, and material packaged within a double-membrane structure through the process of macroautophagy (referred to as autophagy hereafter) (1, 2). The proteasome, as predetermined by its structure, is a protease with different requirements for its substrates (3, 4). The proteolytic active sites of the proteasome are housed inside a cylindrically shaped core particle (CP^3^ or 20S) that is formed by four heptameric rings (α_1–7_, β_1–7,_ β_1–7_, and α_1–7_). Both faces of the CP cylinder are identical and have a regulated entry site known as the gate. Folded polypeptides are too large to enter this gate. Hence, functional proteasomes require one or two regulatory particles (RPs or 19S). The RP provides several important functions in the degradation of substrates as follows: docking of RP on CP opens the gate, RP contains receptors that recognize the substrates (normally ubiquitin modifications found on proteins destined for degradation), and RP contains a ring of six AAA-ATPases that help to unfold and translocate substrates through the gate (3). The latter explains why proteasomal degradation is an energy-requiring process. Thus, proteasomes can readily degrade free soluble proteins; however, the degradation of aggregates or protein complexes requires processing by chaperones prior to degradation (4).

The cell has many large molecular complexes. These complexes are often stable and the proteasome might not be readily able to degrade them. Ribosomes are an example of such a large and stable molecular complex. Upon nitrogen starvation, the need for ribosomes is reduced as cells lack resources to produce large amounts of proteins. Furthermore, the degradation of ribosomes can provide crucial nitrogen-containing compounds required for cell survival (5, 6). Consistent with this, ribosomes have been shown to undergo vacuolar degradation upon nitrogen starvation through an autophagy-dependent pathway (6–8). This is a process of selective autophagy, named ribophagy, based on the dynamics of ribosome packaging and the requirement of specific genes that are dispensable for non-selective bulk autophagy (6–8).

Like ribosomes, the proteasome is a stable, abundant, and large protein complex in the cell (3, 9). Several studies have looked at the relationship between autophagy and the proteasome, and increased proteasome levels and activity have been observed upon inhibition of autophagy (10–14). Nevertheless, the fate of proteasomes under starvation conditions is unclear, with only one report identifying proteasomes in the lysosome of starved rat livers (15). As a large and abundant protein complex, it can provide a substantial source of nutrients. Furthermore, a subset of proteasomes seem to work together with ribosomes. Thus, like ribosomes, proteasomes may be targeted for degradation upon starvation. However, proteasomes can degrade proteins and could, like vacuoles, be important in degrading proteins upon starvation (16).

In this study, we characterized how nitrogen starvation, but not glucose starvation, induces autophagy of proteasomes. Our data indicate that the vacuolar targeting of proteasomes requires nuclear export of proteasomes that are targeted for autophagy. Furthermore, CP and RP are targeted to the vacuole through different mechanisms, as only CP autophagy depends on the deubiquitinating enzyme Ubp3. Thus, our data show the autophagic removal of proteasomes from cells upon nitrogen starvation is not simple non-selective bulk autophagy but is a highly regulated process.

## Experimental Procedures

### 

#### 

##### Yeast Strains

All strains used in this study are reported in Table 1 and were generated in a SUB61 background (*lys2-801 leu2-3, 2-112 ura3-52 his3*-Δ*200 trp1-1*) (17). Strains deleted for specific genes or containing C-terminal GFP fusions of Rpn1 and Pre2 (β5) were generated using standard PCR-based procedures (see Table 2) (18, 19).

**TABLE 1:** **Strain list** All strains have the DF5 background genotype (*lys2-801 leu2–3, 2–112 ura3-52 his3-*Δ*200 trp1-1*). All strains used are from this work.

Strain	Genotype (*lys2-801 leu2-3, 2-112 ura3-52 his3-*Δ*200 trp1-1*)	Fig.
sJR786	MATα rpn1::RPN1-GFP (HIS) atg7::CloNAT	3, *A* and *B*
sJR858	MATα pre2::PRE2-GFP (HIS)	1–6 and 6*D*
sJR861	MATα rpn1::RPN1-GFP (HIS)	1–6 and 6*D*
sJR869	MATα rpn11::RPN11-GFP (HIS)	1, *A* and *C*
sJR875	MATα pre2::PRE2-GFP (HIS) pep4::CloNAT	2, *A*–*C*
sJR876	MATα rpn1::RPN1-GFP (HIS) pep4::CloNAT	2, *A*–*C*
sJR877	MATα rpn1::RPN1-GFP (HIS) nvj1::HYG	3*C*
sJR878	MATα pre2::PRE2-GFP (HIS) nvj1::HYG	3*B*
sJR879	MATα rpn1::RPN1-GFP (HIS) ubp3::HYG	5, *A* and *C*
sJR880	MATα pre2::PRE2-GFP (HIS) ubp3::HYG	5, *B* and *D*
sJR881	MATα pre2::PRE2-GFP (HIS) atg17::HYG	3*D*, and 4, *A* and *B*
sJR882	MATα rpn1::RPN1-GFP (HIS) atg17::HYG	3*C* and 4*A*
sJR883	MATα rpn1::RPN1-GFP (HIS) atg19::HYG	3*C*
sJR889	MATα rpn1::RPN1-GFP (HIS) ufd3::HYG	6, *A* and *C*
sJR890	MATα pre2::PRE2-GFP (HIS) ufd3::HYG	6*B*
sJR896	MATα pre2::PRE2-GFP (HIS) rpn10::HYG	6*D*
sJR897	MATα rpn1::RPN1-GFP (HIS) rpn10::HYG	6*D*
sJR900	MATα rpn1::RPN1-GFP (HIS) atg11::HYG	3*C*
sJR904	MATα pre2::PRE2-GFP (HIS) atg11::HYG	3*D*
sRJ925	MATα pre2::PRE2-GFP (HIS) atg7::CloNAT	3, *A* and *B*

**TABLE 2:**
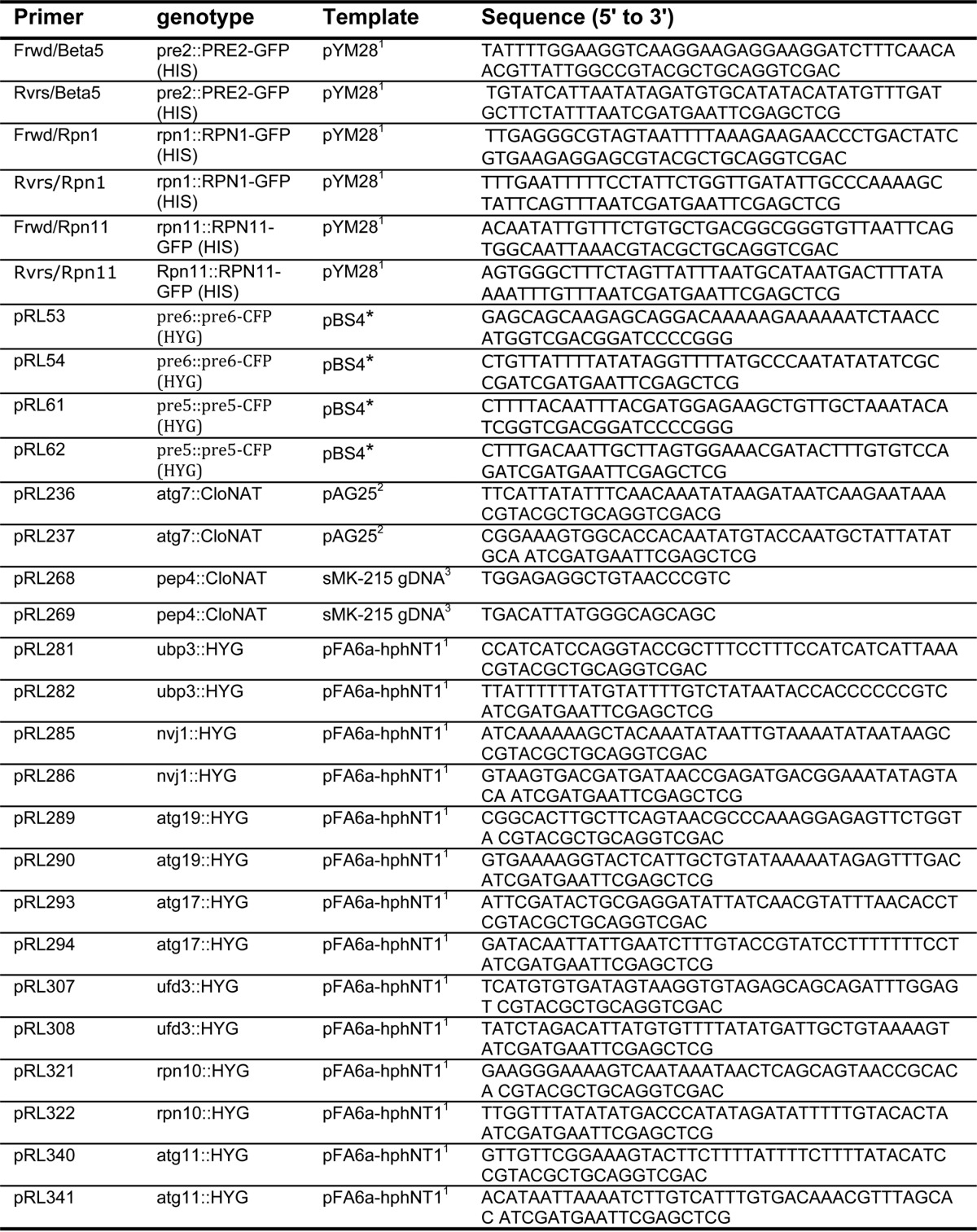
**Primer list**

^1^ See Ref. 19.

^2^ See Ref. 18.

^3^ See Ref. 65.

* Plasmids were generously donated by The Yeast Resource Center at the University of Washington.

##### Yeast Growth Conditions

Yeast cultures were grown in YPD medium or synthetic defined (SD) medium containing 0.17% yeast nitrogen base, 0.5% (NH_4_)_2_SO_4_, 2% dextrose and supplemented with appropriate nutrients (specific amino acids, uracil, and adenine). For starvation, overnight cultures of yeast strains were diluted to an *A*_600_ of 0.5 and grown in YPD medium to an *A*_600_ between 1 and 2. Next, cells were washed with sterile water and resuspended in SD medium at an *A*_600_ of 1.5. For nitrogen starvation, nutrients and ammonium sulfate were omitted from the SD medium; for glucose starvation dextrose was omitted, and for the combined starvation of nitrogen and glucose nutrients, ammonium sulfate, and dextrose were omitted. Cells were grown at 30 °C with constant shaking and harvested at the indicated time points by centrifugation. Cell pellets were stored at −80 °C until processing.

##### Yeast Phenotype

Strains were grown to an *A*_600_ of 1 in YPD. 1 ml of cells was then harvested and washed with sterile water. Serial dilutions of the cells were made in 96-well plates, and cells were transferred to YPD plates or plates containing 0.2 μg/ml rapamycin. Plates were incubated for 24–48 h at 30 °C.

##### Immunoblotting Analysis

Protein lysates were obtained by NaOH/TCA precipitation (6). In short, cell pellets were resuspended in 200 mm NaOH and incubated on ice for 10 min. Trichloroacetic acid was added to a final dilution of 5%, and the mixture was allowed to incubate on ice for 10 min. Cells were pelleted by centrifugation at 18,000 × *g* for 2 min. Pellets were washed with ice-cold acetone and centrifuged at 18,000 × *g* for 2 min. Next, pellets were resuspended in SDS-PAGE sample buffer and boiled at 96 °C for 5 min. Samples were centrifuged at 18,000 × *g* for 5 min, and supernatant was collected. Samples were separated by SDS-PAGE and analyzed by immunoblotting using antibodies against GFP (Roche Applied Science, catalog no.11814460001) and Pgk1 (Invitrogen, catalog no.459250). All images were acquired using a Gbox imaging system (Syngene) with GeneSnap software. Quantifications were carried out by determining the peak volumes for the different bands using the GeneTools analysis software from Syngene. Values were corrected for loading using the Pgk1 signal. Data are displayed as value of free GFP (see under “Results” for definition) divided by the sum of the value of free and subunit associated GFP and are plotted over time. Data shown represent at least three independent biological replicates.

##### Fluorescence Microscopy

Live yeast cells expressing either Rpn1-GFP or β5-GFP with different mutations in these backgrounds were washed with sterile water either before or after starvation. To stain vacuoles, 0.4 μm FM 4-64 was added to logarithmically growing cells 2 h prior to starvation. Cells were transferred to an l-lysine-coated slide, and the coverslip was sealed using VALAP (mixture of equal parts of Vaseline, lanolin, and paraffin wax). Images were acquired at room temperature on a Nikon Eclipse TE2000-S microscope at ×600 magnification using a Plan Apo ×60/1.40 objective and the 490/528 nm (GFP images) and 555/617 nm (FM 4-64 images) Sedat Quad filter set (Chroma 86000v2, Bellows Falls, VT) using a CoolSNAP cf camera (Photometrics). The images were collected using Metamorph (Molecular Devices). Images were prepared using Adobe.

##### Succinyl-LLVY-AMC Hydrolysis Assays

Lysis buffer (50 mm Tris-HCl (pH 8.0), 1 mm ATP, 5 mm MgCl_2_, 1 mm EDTA) was added to nitrogen-starved and non-starved cell pellets equal to pellet volume. Cells were lysed by a combination of freeze-thaw and pestle grinding (at least three rounds) in a 1.5-ml microcentrifuge tube in the presence of liquid nitrogen. Lysates were spun at 18,000 × *g* at 4 °C for 5 min, and the supernatant was collected. Protein concentrations were obtained by NanoDrop, and equal amounts were loaded in a well of a black 96-well plate (Greiner Bio-One, 655086) supplemented with buffer 2 (50 mm Tris-HCl (pH 7.5), 1 mm ATP, 5 mm MgCl_2_, 1 mm EDTA) to a total volume of 50 μl. 50 μl of substrate buffer (buffer 2 supplemented with 200 μg/ml LLVY-AMC (Bachem, I-1395)) was added to the wells to start the reactions. Reactions were followed by measuring fluorescence (excitation 360 nm, 460 nm detection) using a Wallac Victor^2^ microplate reader (PerkinElmer Life Sciences) with plates at 30 °C. Data were normalized for input and displayed as relative fluorescent units.

##### Native Gels

To look at the incorporation of tagged subunits into proteasomes, native gel analyses, including in-gel LLVY-AMC hydrolysis activity, were carried out as described previously (20). Cells were lysed by the liquid nitrogen method described above, and equal amounts of protein, 170 μg, were loaded onto gels. Gels were run for 3.5 h at 4 °C at 100 V and were scanned on Typhoon 9410 imager (excitation 488, 526SP filter).

## Results

### 

#### 

##### Vacuolar Targeting of Proteasomes upon Nitrogen Starvation

**FIGURE 1.**
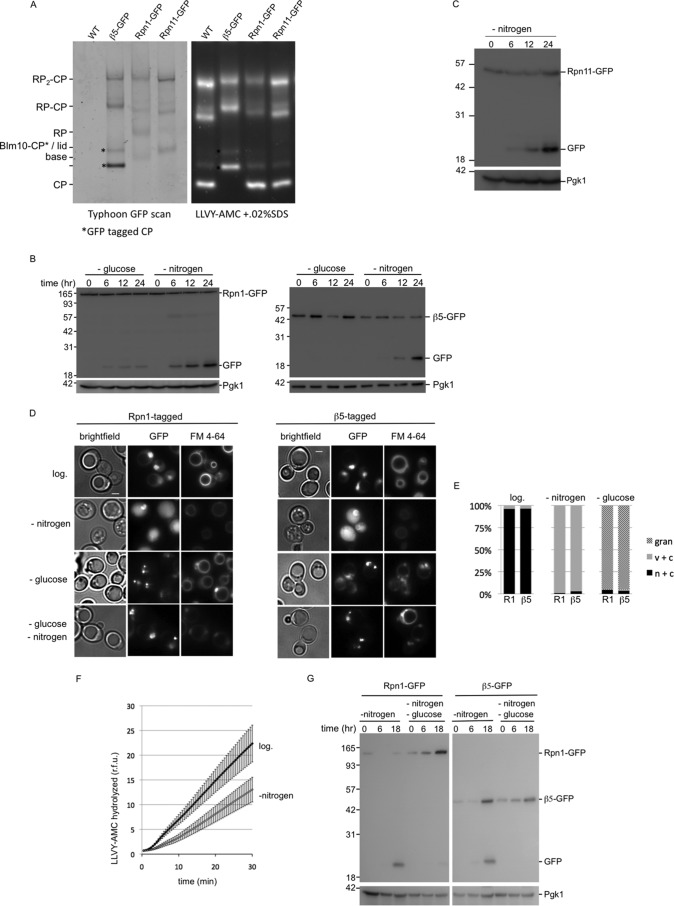
**Nitrogen starvation targets proteasomes to the vacuole.** *A,* strains were C-terminally tagged with GFP at the endogenous locus of the core particle subunit β5, the regulatory particle subunit Rpn1, or the regulatory particle subunit Rpn11. Cell lysates of indicated strains were resolved on native gel and scanned to determine incorporation of GFP-tagged subunits in proteasome complexes. Next, gels were stained with proteasome fluorogenic substrate LLVY-AMC, to visualize proteasome peptidase activity. * indicates CP with β5-GFP; GFP-tagged CP showed a migrational shift compared with untagged CP. The different subunits can be detected in assembled proteasomes and the appropriate subcomplexes (*i.e.* β5 in CP; Rpn1 in RP and base; and Rpn11 in RP and lid). *B,* strains expressing Rpn1-GFP or β5-GFP were grown logarithmically in YPD to *A*_600_ 1.5, washed, and transferred to minimal media lacking either glucose or nitrogen. Cells were collected and lysed at indicated time points. Samples were resolved on SDS-PAGE and immunoblotted for GFP and the loading control Pgk1. *C,* strains expressing Rpn11-GFP were analyzed upon nitrogen starvation as described in *B. D,* strains expressing Rpn1-GFP or β5-GFP were analyzed by fluorescent microscopy for GFP localization prior to starvation or after incubation for 24 h in minimal media lacking glucose or nitrogen. Cells were stained with FM 4-64 to visualize vacuoles. Representative images are shown, and quantifications are shown in *E. E,* at least 100 cells were used to quantify the localization as displayed in the stacked *bar graph* as percentage. *n* + *c*, nuclear and cytosolic staining with stronger signals in nucleus; *c* + *v,* little nuclear staining and clear vacuolar localization; *gran*, presence of distinct granular localization sometimes combined with weak nuclear and cytosolic staining but no vacuolar staining. Three independent experiments showed similar amounts and localization results. *F,* cells were lysed before and after nitrogen starvation. Equal amounts of protein were assayed for hydrolysis of LLVY-AMC. Accumulation of fluorescent AMC over time is displayed as relative fluorescence units (*r.f.u.*). The average of six independent experiments are shown with S.E. Activity upon starvation is reduced to 58% and the difference was highly significant (paired Student's *t* test *p* = 0.01, *n* = 6). *G,* experiment as *B* only cells were starved in minimal media lacking nitrogen or nitrogen and glucose. Localization of the fluorescent signal in double-starved cells (see *D*) resembled that of glucose starvation, showing granules and little to no vacuolar localization.

To test whether proteasomes are targeted for vacuolar degradation upon starvation, we generated strains with a gene fusion that encodes Rpn1-GFP to monitor RP as well as strains with a gene fusion that encodes Pre2-GFP (β5-GFP) to monitor CP. The GFP-encoding sequence was introduced into a genome at the endogenous locus to encode C-terminally GFP-tagged versions of the proteasome subunits Rpn1 and β5, thereby ensuring that 100% of the subunits in the cell are GFP-tagged. Furthermore, the use of the endogenous promoter in its normal genomic environment minimizes artifacts resulting from abnormal expression levels. Both β5 and Rpn1 are essential, and the successful integration of the GFP indicates that these GFP-tagged versions are functional and incorporated into the proteasome. We also confirmed this by using native gel analyses (Fig. 1*A*). Furthermore, these subunits have been successfully tagged at the C terminus and shown to be incorporated into the proteasome in previous studies as well (21–24). Strains with GFP-tagged proteasome subunits allow us to monitor vacuolar targeting of proteasomes by observing the accumulation of a band of ∼25 kDa that is recognized by the GFP antibody on immunoblots. In general, the appearance of this band (referred to as free GFP hereafter) results from rapid cleavage of the linker between GFP and the protein or the protein complex of interest (25, 26). The fold of the GFP protein resists fast vacuolar degradation resulting in the accumulation of free GFP (25, 26). We observed that both Rpn1-GFP and β5-GFP strains accumulate free GFP upon nitrogen starvation (Fig. 1*B*). Using this same approach, we also observed the appearance of free GFP for Rpn11-GFP, indicating these results reflect a common property of proteasomes and not a unique feature of specific proteasome subunits (Fig. 1, *A* and *C*). Microscopic analyses of the Rpn1-GFP- or β5-GFP-expressing strains showed that, prior to starvation, the GFP signal is mainly nuclear (Fig. 1*D*, *top row,* and Hoechst 33342 colocalization, data not shown). This is consistent with reports that proteasomes in yeast are abundant in the nucleus (27, 28). Staining vacuolar membranes with FM 4-64 further revealed that the GFP signal was absent from vacuoles, indicating little or no proteasomes are in the vacuole. Upon nitrogen starvation, the majority of cells lost the nuclear enrichment of the GFP signal with a concurrent appearance of the GFP signal in vacuoles (Fig. 1, *D* and *E*). Please note that FM 4-64 staining was conducted prior to starvation and that in cells with active autophagy the FM 4-64 stain looses brightness over time. Nevertheless, staining still allowed us to unambiguously identify vacuoles. The observed vacuolar targeting is specific for nitrogen starvation, because glucose starvation did not result in accumulation of GFP on immunoblots (Fig. 1*B*). Microscopic analyses showed that glucose starvation does result in relocalization of proteasomes (Fig. 1, *D* and *E*). However, vacuoles remained devoid of GFP signal under these conditions, and the signal was mostly concentrated in distinct structures that appear perinuclear. This localization is consistent with previous reports that show the translocation of proteasomes from the nucleus and cytosol into proteasome storage granules (PSGs) upon glucose starvation or upon entering into stationary phase (29, 30). Thus, proteasomes relocalize upon starvation; nitrogen starvation leads to vacuolar localization and glucose starvation to PSGs.

If nitrogen starvation results in vacuolar targeting of proteasomes, one would predict a reduced proteasome activity in lysates upon nitrogen starvation. We tested this by comparing peptidase activity in nitrogen-starved and non-starved cells using LLVY-AMC as substrate. Upon starvation, we observed strong reduction in LLVY-AMC peptidase activity (∼40–50%). For both starved and non-starved conditions, the addition of the proteasome inhibitor bortezomib (50 μm) reduced the peptidase activity to less than 3.5% (data not shown), indicating that the activity represents proteasome peptidase activity. The reduction in peptidase activity upon starvation is consistent with previous observations and with proteasomal targeting to the vacuole (Fig. 1*F*) (10).

Next, we tested whether cells starved for both nitrogen and glucose showed vacuolar targeting of proteasomes (Fig. 1, *D* and *G*). Surprisingly, in the presence of both starvation conditions, proteasomes do not undergo vacuolar targeting, but appear to end up in PSGs (Fig. 1*D*). However, this process seems less efficient or somewhat delayed compared with only glucose starvation. Thus, nitrogen starvation does not always lead to vacuolar targeting of proteasomes, as it depends on the physiological state of the cells.

**FIGURE 2.**
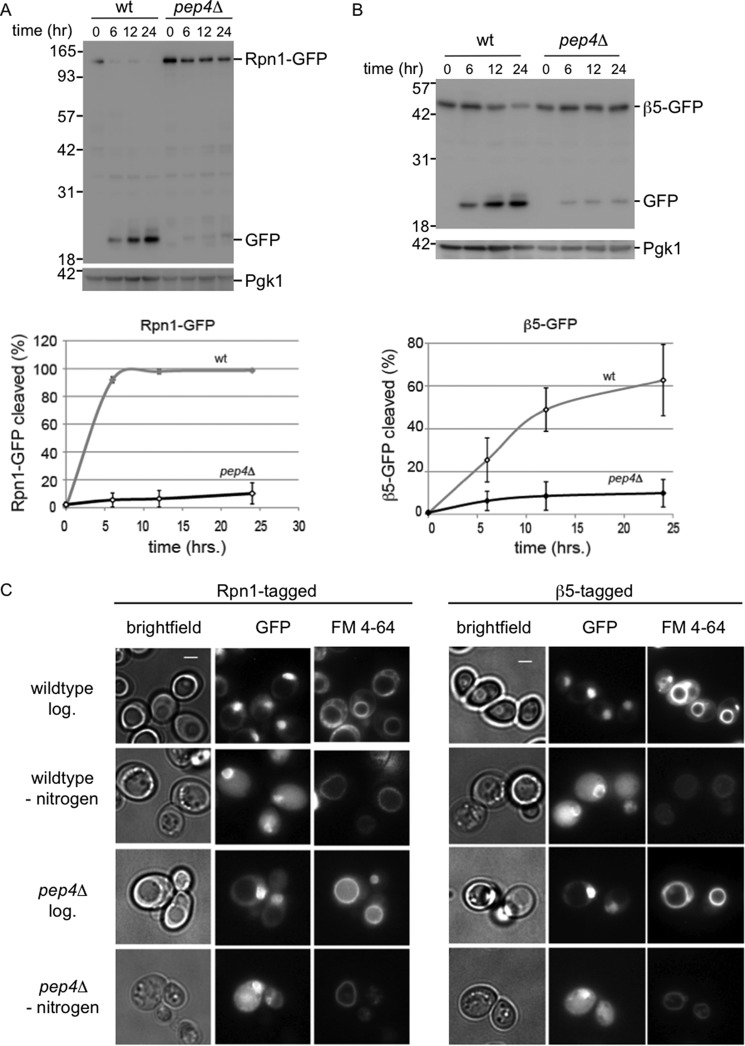
**Vacuolar hydrolases are responsible for cleavage of tagged proteasome subunits.** *A* and *B,* wild type or *pep4*Δ strains with gene fusions encoding Rpn1-GFP (*A*) or β5-GFP (*B*) were starved for nitrogen in the presence of 1 mm PMSF and lysed. Samples were analyzed as in Fig. 1*C*. Three independent immunoblots were quantified and corrected for input (Pgk1 amounts), and percentage cleaved (displayed on *y* axis) was calculated as amount of Rpn1-GFP signal divided by the amount of Rpn1-GFP signal plus free GFP signal for each time point. *C,* cells were analyzed by fluorescent microscopy to visualize proteasome subunits (GFP) and vacuoles (FM 4-64) prior to starvation or after incubation for 24 h in minimal media lacking nitrogen.

To confirm that the processing of GFP-tagged proteasomes upon nitrogen starvation occurs in the vacuole, we tested the dependence of this processing on acidic hydrolases using a strain deleted for *PEP4*. The deletion of *PEP4* in combination with the addition of the protease inhibitor PMSF cause a strong reduction in hydrolytic activity by vacuolar hydrolases, as many hydrolases depend on Pep4 for maturation (31). The strongly reduced levels of free GFP in these strains confirm that, indeed, the free GFP appears as a result of the activity of vacuolar acidic hydrolases (Fig. 2, *A* and *B*). Microscopic analyses of strains deleted for *PEP4* showed that proteasomes are still targeted to the vacuole in these cells, and consequently, images are similar to those observed for the wild type strain after nitrogen starvation (Fig. 2*C*).

##### Vacuolar Targeting of Proteasomes Depends on the Autophagy Pathway

**FIGURE 3.**
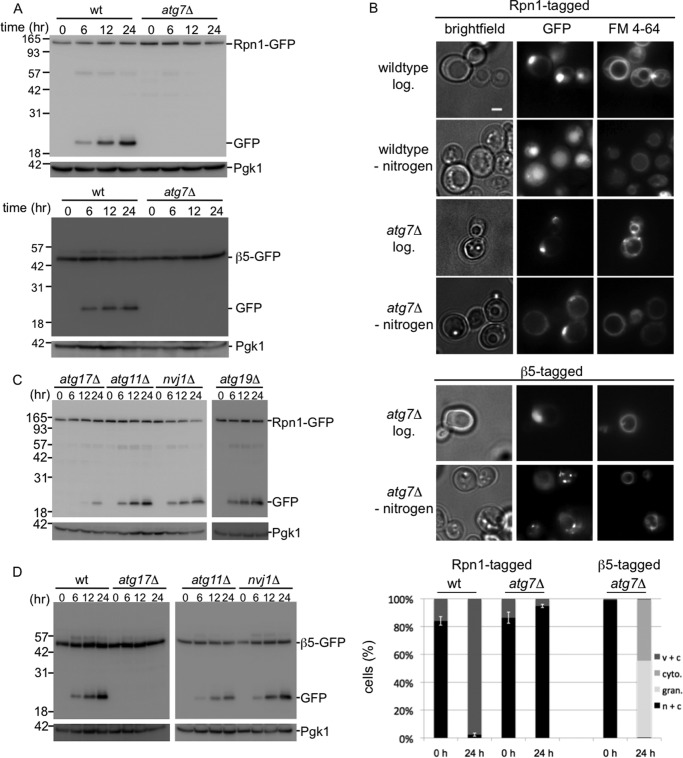
**Proteasomes are targeted to the vacuole by autophagy.** *A,* wild type or *atg7*Δ strains with gene fusions encoding Rpn1-GFP or β5-GFP were starved for nitrogen and lysed. Samples were resolved on SDS-PAGE and immunoblotted for GFP and the loading control Pgk1. *B,* microscope images and quantification (*n* > 100) of cells as in *A. n* + *c,* nuclear and cytosolic staining with stronger signals in nucleus; *c* + *v*, little nuclear staining and clear vacuolar localization; *cyto*, clear cytosolic staining without nuclear enrichment and no vacuolar staining; *gran*, presence of distinct granular localization sometimes combined with weak nuclear and cytosolic staining but no vacuolar staining. Three independent experiments showed similar amounts and localization results. *C* and *D,* gene fusions encoding Rpn1-GFP (*C*) or β5-GFP (*D*) were introduced in strains deleted for indicated genes and analyzed as in *A*.

Recent work in yeast suggests that glucose starvation inhibits autophagy and leads to endocytosis of substantial amounts of membrane proteins and lipids (32). Nitrogen starvation, however, is well known to induce autophagy (1). Thus, the most likely pathway for the proteasome to be targeted to the vacuole is autophagy. A common core of autophagy genes is required for all autophagy. However, there are several forms of selective autophagy, each requiring a specific subset of additional factors (1, 2). To test the involvement of autophagy genes in this process, we first deleted the *ATG7* gene from Rpn1-GFP- and β5-GFP-tagged strains. Atg7 is crucial for macro- and microautophagy; it is a ubiquitin-activating enzyme homolog that activates Atg12 and Atg8, two polypeptides that ultimately are attached to Atg5 and phosphatidylethanolamine, respectively, in the autophagic process (1). No free GFP was detected, and the GFP remained covalently attached to Rpn1 and β5 upon nitrogen starvation in the *atg7*Δ cells (Fig. 3*A*). Microscopic analyses of these cells shows that, in the absence of *ATG7,* the fluorescent signal does not accumulate in the vacuole, indicating that proteasomes are not transported to the vacuole (Fig. 3*B*). Thus, the vacuolar targeting of proteasomes depends on a functional autophagy pathway. Next, we tested the need for the macroautophagy core component Atg17 (Fig. 3, *C* and *D*) (33). The deletion of *ATG17* in the Rpn1-GFP background resulted in a strong reduction in the formation of free GFP after nitrogen starvation. For β5-GFP, we were unable to detect any free GFP in this background. Thus, Atg17 plays an important role in autophagy of proteasomes. The small amount of free GFP observed in the Rpn1-GFP strain might indicate the presence of a minor Atg17-independent pathway, because we never observed any free GFP when analyzing Rpn1-GFP *atg7*Δ strains. Atg17 is an early acting protein involved in the formation of the preautophagosomal structure (PAS), which is essential for the formation of autophagosomes upon starvation (33). Nevertheless, it has also been reported to play a role in piecemeal microautophagy (34). Piecemeal autophagy is a process in which nuclear envelope material as well as nuclear content are directly targeted to the vacuole for degradation. Considering the nuclear localization of a large portion of proteasomes under logarithmic growth (Fig. 1*D*) (27, 28), Atg17 might impact proteasome autophagy through this pathway. The piecemeal autophagy pathway depends on the nuclear envelope receptor Nvj1 (35). However, the autophagy of neither CP nor RP was substantially reduced upon deletion of *NVJ1* (Fig. 3, *C* and *D*). Thus, proteasomes are not targeted for degradation through Nvj1-dependent piecemeal autophagy and are thus most likely exported out of the nucleus prior to autophagic packaging. Cytosolic protein material in yeast can be targeted to the vacuole through a specialized form of autophagy called the cytosol-to-vacuole targeting pathway (1). To test whether proteasome autophagy depends on the cytosol-to-vacuole targeting pathway, we deleted *ATG19* in an Rpn1-GFP-tagged strain, which impairs this process. Immunoblots from nitrogen-starved samples showed that proteasome autophagy still occurs at high efficiency in strains impaired in the cytosol-to-vacuole targeting pathway (Fig. 3*C*). Thus, proteasome autophagy does not depend on this yeast-specific pathway to transport proteasomes to the vacuole but requires *ATG7* and *ATG17,* genes that are conserved between yeast and humans.

Many forms of selective autophagy depend on the adaptor protein Atg11, which interacts with various receptors (2, 36). Furthermore, Atg11 can interact with Atg17. To our surprise, *ATG11* deleted cells were still able to efficiently perform nitrogen-induced proteasome autophagy, as both Rpn1-GFP- and β5-GFP-tagged strains showed free GFP upon nitrogen starvation in an *atg11*Δ background (Fig. 3, *C* and *D*). Thus, this adaptor, which is common to many forms of selective autophagy, is not required for targeting this multisubunit protein complex for autophagy. This might suggest that proteasome autophagy is not selective or reflects a more general difference between autophagy of organelles (many require Atg11) and protein complexes as the autophagy of acetaldehyde dehydrogenase (and presumably ribosomes as well) also requires Atg17 but is independent of Atg11 (2, 37, 38). Autophagy of leucine aminopeptidase III (Lap3) depends on Atg17 and to a lesser extent on Atg11. However, Lap3 shows some Atg17-independent autophagy similar to Rpn1 (Fig. 3*C*) (37).

##### RP and CP Are Processed Independently for Proteaphagy

**FIGURE 4.**
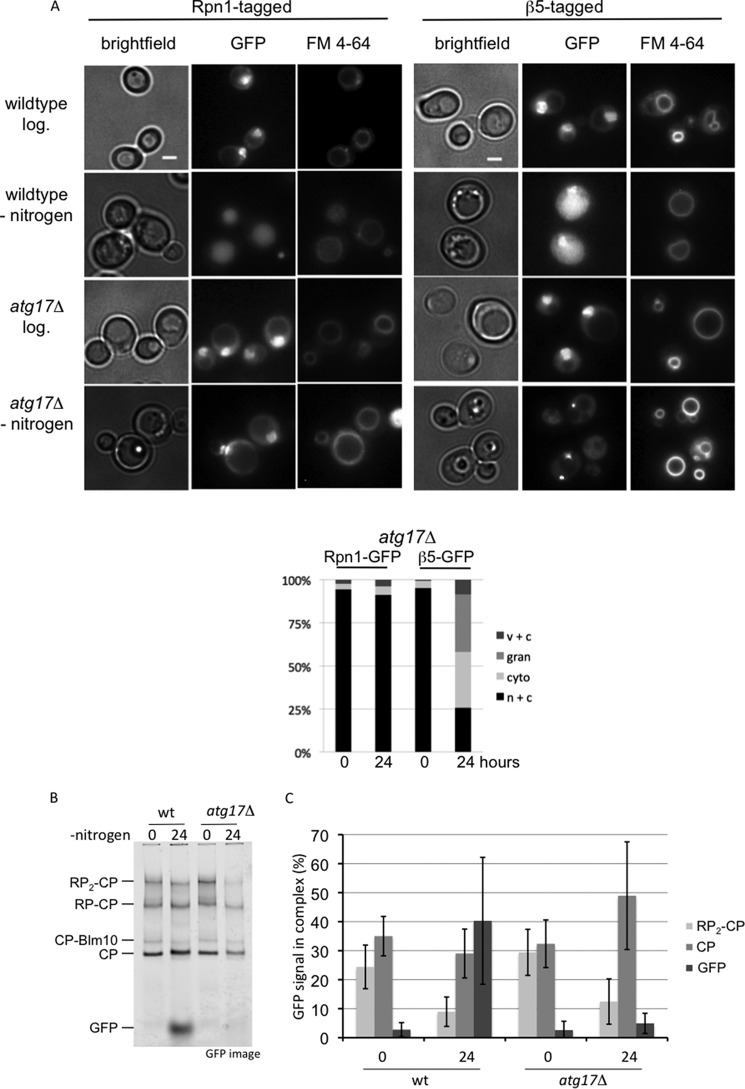
**Different processing of proteasome CP and RP autophagy.** *A,* microscope images and quantification (*n* > 100) of *atg17*Δ cells. *n* + *c*, nuclear and cytosolic staining with stronger signals in nucleus; *c* + *v*, little nuclear staining and clear vacuolar localization; *cyto*, clear cytosolic staining with little to no vacuolar or nuclear staining; *gran*, presence of distinct granular localization sometimes combined with weak nuclear and cytosolic staining but no vacuolar staining. Note that for Rpn1-GFP after nitrogen starvation in the *atg17*Δ cells, the nuclear to cytosol ratio becomes somewhat smaller, but it remains enriched in the nucleus and hence no difference in our scoring. *B,* wild type and *atg17*Δ cells expressing β5-GFP were lysed, and samples were resolved on native gel. Gels were scanned on Typhoon 9410 imager to identify the complexes in which β5-GFP was incorporated. *C,* quantification of native gels from *B*. For each condition, the percentage of β5-GFP in a particular proteasome complex was determined. Displayed is the average of three independent experiments. *Error bars* indicate S.E.

Microscopic analyses of the localization of Rpn1-GFP and β5-GFP upon nitrogen starvation in strains deleted for *ATG7* or *ATG17* showed a remarkable difference; Rpn1-GFP remained enriched in the nucleus, although a substantial amount of β5-GFP accumulated in the cytosol and granules resembling PSGs (Figs. 3*B* and 4*A*). This suggests that nitrogen starvation leads to some dissociation of proteasomes into RP and CP complexes. To test this, we analyzed wild type and *atg17*Δ strains expressing β5-GFP on native gels before and after nitrogen starvation (Fig. 4*B*). As the protein content of cells under the different conditions might be different per cell, and we loaded equal amounts of proteins on the gel, we analyzed the distribution of β5-GFP among the different complexes as percentage of total β5-GFP for each sample, using the average results of three independent experiments (Fig. 4*B*). For wild type cells, nitrogen starvation results in a dramatic reduction in the percentage of assembled proteasomes (RP_2_-CP species went from 24 to 9%). Concurrent with a reduction in 26S proteasomes, there was a dramatic increase in free GFP (3–40%). Upon starvation of *atg17*Δ cells, the amount of assembled proteasomes also goes down (RP_2_-CP from 29 to 12%). However, instead of an increase in free GFP, now the levels of CP are increased (32–49%). These data show that β5-GFP is monitoring what happens to proteasomes and CP and does not show artifacts resulting from an unincorporated subunit. Furthermore, these data show that nitrogen starvation leads to a substantial amount of dissociation of proteasomes even if autophagy is blocked.

**FIGURE 5.**
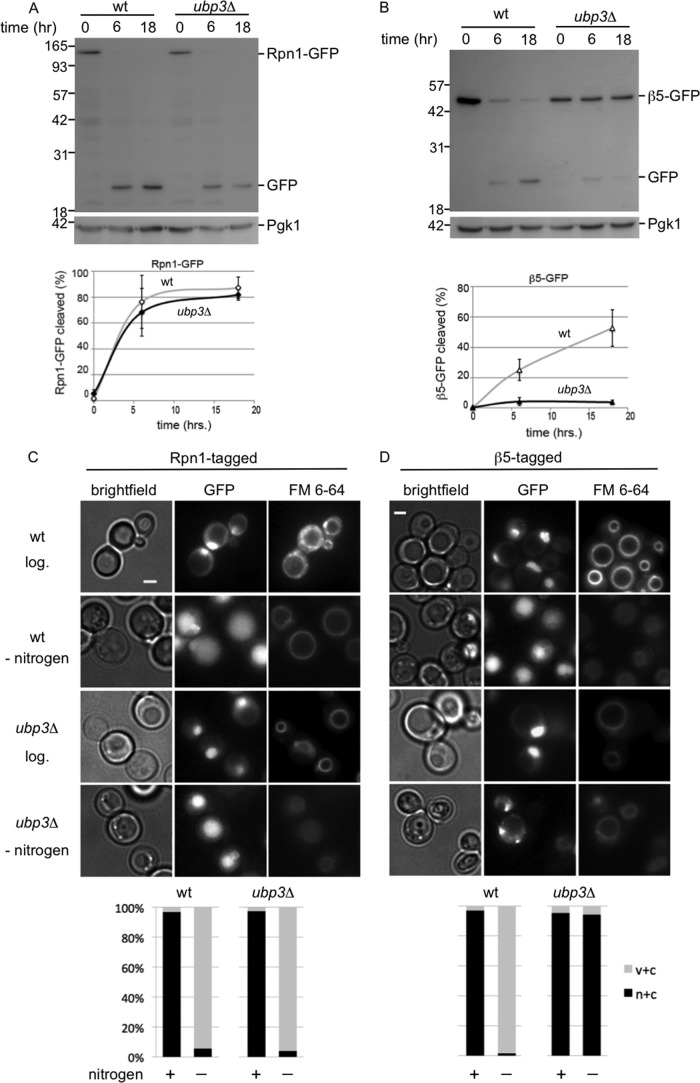
**Proteasome core particles and regulatory particles depend differently on Ubp3 for vacuolar targeting.** *A* and *B,* wild type or *ubp3*Δ strains with gene fusions encoding Rpn1-GFP (*A*) or β5-GFP (*B*) were analyzed for vacuolar targeting of RP and CP, respectively, upon nitrogen starvation. Samples were resolved on SDS-PAGE and immunoblotted for GFP and the loading control Pgk1. *Lower panel* shows quantifications as in Fig. 2. Shown are the averages from 3 to 5 experiments, and *error bars* indicate S.E. *C* and *D,* microscope images and quantification (*n* > 100) of cells from *A* and *B* before and after 24 h of starvation. *c* + *v*, little nuclear staining and clear vacuolar localization; *n* + *c*, nuclear and cytosolic staining with stronger signals in nucleus, except for β5-GFP *ubp3*Δ cells where there was clearly stronger signal in the cytosol. Irrespectively, all *n* + *c* all have little to no vacuolar staining.

Additional data supporting the notion that CP and RP are targeted for autophagy separately came from our observation that RP and CP are affected differently by a deletion of *UBP3*. Ubp3 is a deubiquitinating enzyme important for ribophagy of the 60S ribosome but interestingly not the 40S ribosome (6). We studied how the deletion of *UBP3* impacts the vacuolar targeting of Rpn1-GFP and observed no difference in accumulated free GFP in *ubp3*Δ compared with wild type cells (Fig. 5*A*). This indicates that the deubiquitinating enzyme Ubp3 is not necessary for targeting Rpn1-GFP and presumably the complete RP subcomplex to the vacuole. However, β5-GFP showed strongly impaired vacuolar targeting, indicating the targeting of CP to the vacuole is dependent on Ubp3 (Fig. 5*B*). Consistent with the immunoblots, microscopic analyses showed the absence of vacuolar GFP signals for β5-GFP-expressing strains in the *ubp3*Δ background but not Rpn1-GFP-expressing strains (Fig. 5, *C* and *D*). Thus, the targeting of the proteasome core particle for autophagy depends on Ubp3.

**FIGURE 6.**
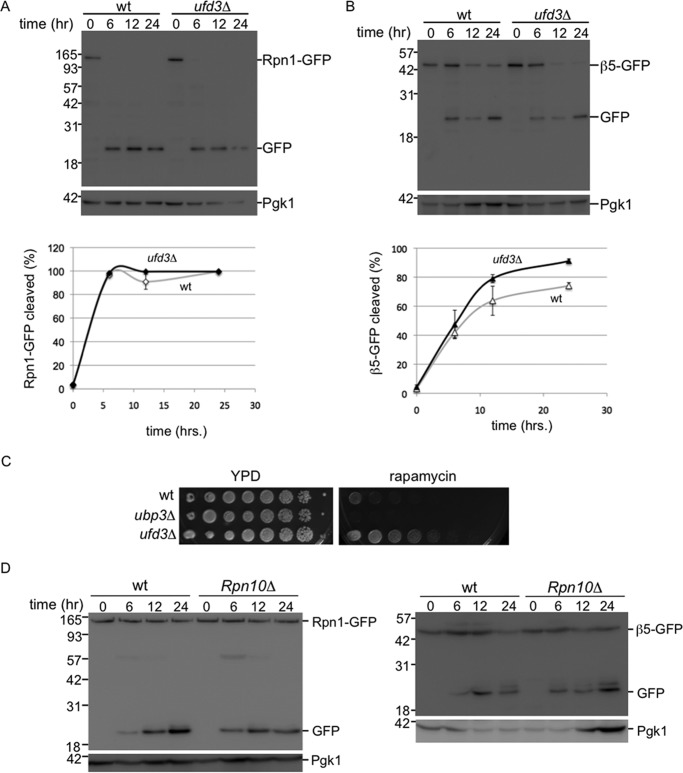
**Proteaphagy does not depend on Ufd3 or Rpn10.** *A* and *B,* CP proteaphagy is distinct from 60S ribophagy as it does not depend on Ufd3. Strains expressing Rpn1-GFP (*A*) or β5-GFP (*B*), either in wild type or *UFD3* deletion background, were starved for nitrogen and lysed. Samples were resolved on SDS-PAGE and immunoblotted for GFP and the loading control Pgk1 (*top panel*). *Lower panel* shows quantification of three independent experiments. *Error bars* indicate S.E. *C,* strains were plated in 4-fold serial dilution on indicated plates. *D,* nitrogen starvation induced proteaphagy does require Rpn10. Indicated strains expressing Rpn1-GFP (*left*) or β5-GFP (*right*) were analyzed as in *A*.

Autophagy of proteasomes shows remarkable similarities with ribophagy; both occur upon nitrogen starvation and have one subcomplex that requires Ubp3 (CP and 60S), although the other does not (RP and 40S). To test whether the degradation of proteasomes and ribosomes is completely dependent on the same machinery, we analyzed whether proteaphagy required Ufd3 (also known as Doa1), a protein known to be necessary for ribophagy (7). Ufd3 is a cofactor of Cdc48/p97 that can bind ubiquitin. Neither CP nor RP required Ufd3 for efficient autophagy (Fig. 6, *A* and *B*), indicating that ribophagy and proteaphagy are distinct processes that share a subset of molecular components for targeting these complexes to the vacuole. A broader role of Ubp3 in autophagy beyond ribophagy, is also suggested from our phenotypic analysis (Fig. 6*C*). Previously, it had been argued that sensitivity of *ubp3*Δ cells to nitrogen starvation and rapamycin indicates the importance of ribophagy for cell survival under starvation conditions. If true, *ufd3*Δ, which is also important for ribophagy (7), should show similar sensitivity to starvation as *ubp3*Δ cells. However, our analysis shows *ufd3*Δ resistance to rapamycin, although *ubp3*Δ is sensitive (Fig. 6*C*). Thus, the role of Ubp3 in starvation and rapamycin responses is likely to be more complex and expand beyond ribophagy. Our observations for a role of Ubp3 in proteasome autophagy is consistent with a broader role of Ubp3.

Because a majority of proteasomes are in the nucleus, it is interesting to analyze how disruption of the autophagy process affects the localization of proteasomes following nitrogen starvation. Here, as briefly mentioned earlier, we observed another difference between CP and RP. Upon disruption of the autophagy process in the Rpn1-GFP background, like the deletion of *ATG7* or *ATG17*, Rpn1-GFP remains largely nuclear upon nitrogen starvation (see *atg7*Δ and *atg17*Δ in Figs. 3*B* and 4*A*). For β5-GFP-tagged strains disrupted in the autophagy process, the β5-GFP signal does not remain largely nuclear and an accumulation of fluorescent signal outside the nucleus was observed (see *atg7*Δ, *atg17*Δ, and *ubp3*Δ in Figs. 3*B*, 4*A*, and 5*D*). In the *atg7*Δ and *atg17*Δ backgrounds, a substantial amount of the β5-GFP signal appears in granule-like structures that show similarity to PSGs (Fig. 4*A*). However, co-localization studies will have to confirm whether these are indeed PSGs. In the *ubp3*Δ background there is no nuclear enrichment upon nitrogen starvation, but granular accumulation is observed more sporadically. In all, these data are consistent with the model that, upon nitrogen starvation, CP and RP dissociate in the nucleus and each is targeted to the lysosome using, at least partly, different pathways.

## Discussion

Proteasomes, like ribosomes, are very stable complexes, and little attention has been given to the process that removes them from cells. Nevertheless, cells need to be able to dispose of proteasomes and other large complexes for several reasons. First, cells need to specifically remove complexes that are not functioning optimally. Non-functional complexes can be present for various reasons. For example, functional complexes can become non-functional as a result of damage, like oxidation, or inhibitor treatment. Interestingly, in *Arabidopsis* it was recently shown that chemically inhibited proteasomes are removed through autophagy in a selective process coined proteaphagy (39). This pathway depended on the proteasome subunit Rpn10, a proteasome subunit not involved in nitrogen starvation-induced autophagy (Fig. 6*D*). Damaged ribosomes have also been proposed to be removed through autophagy (8, 40, 41).

Non-functional complexes can also form during assembly. The assembly of large multisubunit complexes is error-prone as efficient and accurate assembly depends on a number of assembly chaperones (42–46). Misassembled subcomplexes are sometimes recognized by traditional protein quality control machinery, as observed for mutant forms of the proteasome subunit Rpn5 (47). However, it is unlikely that traditional protein quality control mechanisms and the ubiquitin-proteasome system can always recognize and process all forms of misassembled complexes, particularly because they may be composed of properly folded subunits. Consistent with this, assembly of ribosomes and proteasomes has been shown to be under the control of complex-specific quality control mechanisms (40, 43–45, 48). An important requirement for the removal of complexes that are not functioning optimally is the ability to specifically recognize such complexes and target only them for degradation. For proteasomes, the proteasome-associated protein Ecm29 has been shown to specifically bind to certain faulty proteasomes, and it is abundantly present on many mutants, suggesting it might play a role in targeting faulty proteasomes for autophagy (43, 44, 49–51).

A second reason to remove specific complexes from cells is to reduce the overall level of that complex. This can occur when cells encounter conditions that have reduced need for the complex. For example, ribosomes are degraded in the vacuole upon nitrogen starvation (6, 8). We show here that the same is true for proteasomes. Glucose starvation does not induce a similar response, instead proteasomes are transported to PSGs. This could be because the degradation of proteins is not the most efficient source for the energy required, hence the preference for endocytosis of glycosylated material and lipids (32). In contrast, proteins do provide a good source of amino acids and nitrogen compounds that become scarce upon nitrogen starvation. Alternatively, in evolution, glucose starvation might have been a more transient physiological state where cells could benefit from a quick recovery through an ability to retrieve functional proteasomes from the PSGs. Indeed, the addition of glucose to stationary cells has been shown to reverse the effects of glucose starvation with proteasomes going from PSG back to the nucleus (21, 29). If true, however, we would expect the nitrogen response to dominate glucose starvation. Because we observe the opposite, there are likely other factors to consider. Nevertheless, our data presented here show that proteasomes are removed through autophagy upon nitrogen starvation. Here, to reduce proteasome levels, normally functioning proteasomes are likely targeted for degradation, and there is no need to recognize a specific subset of proteasomes.

It is unclear whether nitrogen starvation-induced proteaphagy is a form of selective autophagy, like the proteaphagy of inhibited proteasomes (39). Our data show that nuclear proteasomes are targeted for autophagy upon nitrogen starvation and show that starvation induces a dissociation of CP and RP, indicating that there must be signaling events in the cell that initiate this process. This does not necessarily imply selective autophagy, as the signaling could simply result in nuclear export, after which bulk autophagy engulfs the cytosolic proteasomes for degradation. However, we think this in unlikely for two reasons. First, the blocking of autophagy results in nuclear RP, suggesting there is a link between autophagy and nuclear export. Second, the autophagy of CP depends on Ubp3, a factor known to be dispensable for bulk autophagy. Furthermore, our data show that CP is cytosolic in a *ubp3*Δ strain and thus would be readily available for bulk autophagy. Nevertheless, without the identification of a receptor that links the proteasomes to the PAS, alternative interpretations are possible. For example, it could be that proteasomes are subject to bulk autophagy but that the presence of ubiquitinated CP subunits prevents the CP from being incorporated into autophagosomal structures. A similar mechanism might be at play for ribosomes. Here, no autophagy receptor has been identified, and the deletion of the ubiquitin ligase gene *LTN1* can rescue the ribophagy defects of *ubp3*Δ cells. The latter suggests that the removal of ubiquitin by Ubp3 is not involved in an active signaling event for selective autophagy but removes some inhibitory signal.

**FIGURE 7.**
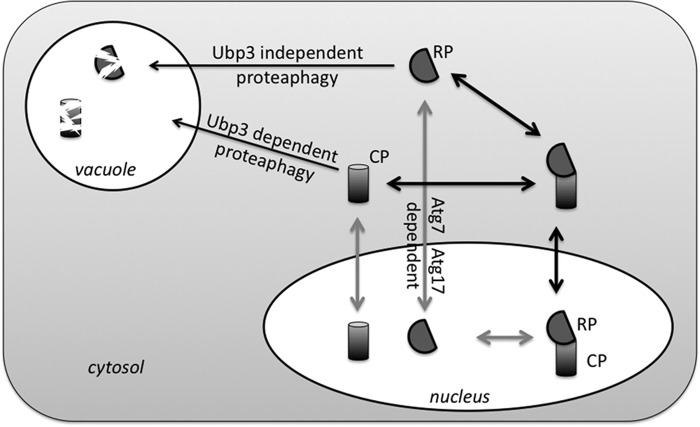
**Proposed model for proteaphagy.** During logarithmic growth ∼70% of yeast proteasomes are nuclear. Nitrogen starvation induces autophagy of proteasomes. However, similar to the observations for ribosomes, two subcomplexes (CP and RP) show a different requirement for components that target them to the vacuole as well as different fates upon disruption of autophagy. This indicates that CP and RP dissociate. It is currently not clear whether this dissociation occurs prior to or following nuclear export resulting in two possible models of nitrogen starvation-induced proteaphagy.

Even if nitrogen starvation-induced proteaphagy is not a form of selective autophagy, the process must involve several specific signaling events, because the majority of proteasomes in yeast are nuclear and not readily available for bulk autophagy. The removal of nuclear components either involves nucleophagy or export from the nucleus prior to autophagy. Based on our observation that *NVJ1*, a gene required for piecemeal autophagy, is not required for proteaphagy, we propose a model where proteasomes are exported from the nucleus prior to autophagy (Fig. 7). Nevertheless, our observation that RP is localized in the nucleus upon blocking of autophagy could indicate some form of nucleophagy is responsible for targeting RP for autophagy. Interestingly, *ATG39* has recently been identified as a receptor in a nucleophagy pathway that did not depend on *NVJ1* (52). However, it is unlikely this pathway is responsible for RP autophagy because Atg39 interacts with Atg11, and RP autophagy is not dependent on *ATG11* (Fig. 3) (52). Thus, unless other forms of nucleophagy exist or are involved (53), RP needs to be exported out of the nucleus prior to autophagic packaging. The nuclear localization of Rpn1-GFP could then indicate a process where RP export and autophagy are linked.

For a long time proteasomes have been considered too large for transport through the nucleopore complex as they enter the nucleus as subcomplexes (22, 54, 55). This suggests that nitrogen starvation (as well as glucose starvation) requires the induction of a signal that leads to RP-CP dissociation prior to nuclear export. This is supported by our observations that a substantial fraction of CP is found in the cytosol in nitrogen-starved *atg7*Δ and *atg17*Δ cells, although RP remains largely nuclear. However, it has recently been shown that 26S proteasomes can be transported through the nuclear pore (23). Thus, proteasomes could be exported as fully assembled complexes and dissociate in the cytosol. In either case, there must be a nitrogen starvation-induced signal that leads to nuclear export and a signal that leads to dissociation of proteasomes. Phosphorylation is a likely candidate as nitrogen starvation is well known to regulate specific kinases (56). Furthermore, ubiquitination seems an obvious candidate. It has been shown to play a role in mitophagy, ribophagy, and proteasome inhibitor-induced proteaphagy among others (39, 57). In proteasome inhibitor-induced proteaphagy, the ubiquitination appears to induce autophagy (39). In contrast, in nitrogen starvation-induced CP proteaphagy, the need for a deubiquitinating enzyme might suggest an opposite role for ubiquitination. However, the deubiquitination might also be important in a late stage to recycle the ubiquitin at the PAS, similar to how Doa4 removes ubiquitin at the multivesicular body (58). Until we identify the modified residue(s) or the E3 enzyme involved, we cannot determine whether an initial ubiquitination followed by a deubiquitination is important for CP autophagy or whether ubiquitination only serves as an inhibitory signal for CP autophagy.

In strains where CP autophagy is blocked, we observed CP in the cytosol upon nitrogen starvation (*ubp3*Δ, *atg7*Δ, and *atg17*Δ) and also a substantial amount in more granule-like structures (*atg7*Δ and *atg17*Δ). It will be interesting to determine whether these structures are reminiscent of PSGs observed after glucose starvation. If so, this could indicate that the initial pathways leading to proteaphagy and PSG formation are shared with respect to proteasome dissociation and nuclear export. This is a strong possibility because there is substantial cross-talk between signaling pathways that regulate autophagy, nitrogen starvation, and glucose starvation (like target of rapamycin complex 1, protein kinase A, and AMP-activated kinase) (56, 59). Interestingly, yeast in a stationary phase, presumably starved for carbon as well as nitrogen, also show proteasomes targeted to PSGs, consistent with our observation during combined starvation (29).

Considering the numerous pathways and stress responses that rely on the degradation by proteasomes, the cellular levels of proteasomes are very important. A number of studies have shown the ability of cells to up-regulate proteasome levels under specific conditions, through Rpn4 in yeast or Nrf1 and Nrf2 in mammalian cells (60–64). In this study, we characterized how nitrogen starvation, but not glucose starvation, induces autophagy of proteasomes in yeast. Starvation-induced proteaphagy has been observed in plants and humans as well, indicating the process is conserved throughout eukaryotic evolution (15, 39). However, our study provides the first indication that an excess of functional proteasomes, *e.g.* as a result of starvation, can be selectively removed through the process of regulated proteaphagy in yeast. This provides a new dynamic component to the regulation of this normally very stable multisubunit complex.

## Author Contributions

The studies were conceived by A. D. M. P., J. R., and K. A. W. K. A. W., A. D. M. P., and G. V. performed the experiments, and all authors analyzed the data. Figures were generated by K. A. W. and A. D. M. P. with input from J. R. The manuscript was written by K. A. W. and J. R. with input from all authors. All authors reviewed the results and approved the final version of the manuscript.

## Footnotes

3 The abbreviations used are:

CP: core particle
PAS: preautophagosomal structures
PSG: proteasome storage granule
RP: regulatory particle
AMC: amido-4-methylcoumarin
SD: synthetic defined.
